# Importance of Lipopolysaccharide and Cyclic **β**-1,2-Glucans in *Brucella*-Mammalian Infections

**DOI:** 10.1155/2010/124509

**Published:** 2010-12-01

**Authors:** Andreas F. Haag, Kamila K. Myka, Markus F. F. Arnold, Paola Caro-Hernández, Gail P. Ferguson

**Affiliations:** School of Medicine & Dentistry, Institute of Medical Sciences, University of Aberdeen, Foresterhill, Aberdeen AB25 2ZD, UK

## Abstract

*Brucella* species are the causative agents of one of the most prevalent zoonotic diseases: brucellosis. Infections by *Brucella* species cause major economic losses in agriculture, leading to abortions in infected animals and resulting in a severe, although rarely lethal, debilitating disease in humans. *Brucella* species persist as intracellular pathogens that manage to effectively evade recognition by the host's immune system. Sugar-modified components in the *Brucella* cell envelope play an important role in their host interaction. *Brucella* lipopolysaccharide (LPS), unlike *Escherichia coli* LPS, does not trigger the host's innate immune system. *Brucella* produces cyclic *β*-1,2-glucans, which are important for targeting them to their replicative niche in the endoplasmic reticulum within the host cell. This paper will focus on the role of LPS and cyclic *β*-1,2-glucans in *Brucella*-mammalian infections and discuss the use of mutants, within the biosynthesis pathway of these cell envelope structures, in vaccine development.

## 1. Introduction

Brucellosis is a disease that can be found in most countries around the world and is transferred from animals to humans [1]. With more than 500,000 new cases of human infections each year, it is the most prevalent zoonotic disease worldwide [1]. Although brucellosis very rarely leads to the death of the patient, it is a seriously debilitating disease that presents with, among other symptoms, fever, fatigue, nausea, and weight loss [2]. Brucellosis is thought to be underreported as the symptoms very often are mistaken for a common flu [2]. However, if not properly treated, brucellosis can become a chronic and asymptomatic disease that can re-emerge months after the initial infection [2]. The causative agents of brucellosis are brucellae, nonmotile, Gram-negative *α*-proteobacteria that are facultative intracellular pathogens [2]. The genus *Brucella *currently contains ten species, named primarily after their preferred host organism or symptoms of the infection: *B. melitensis *(goats and sheep), *B. abortus *(cattle) [3], *B. suis* (swine, reindeer and rodents) [4], *B. canis* (dogs) [5],* B. ovis* (sheep) [6], *B. neomtomae *(rodents) [7],* B. microti* (voles and red foxes) [8], *B. inopinata* (unknown) [9],* B. pinnipedialis *(seals), and *B. ceti* (dolphins and porpoises) [10]. Most human *Brucella *infections can be traced back to the three species, *B. melitensis, B. suis, *and *B. abortus* [11]. The isolation of marine mammal *Brucella *species (*B. pinnipedialis *and *B. ceti*) from human patients, however, suggests that these species are emerging human pathogens [10]. 

Brucellae enter their hosts either through contact with infected animals and material, such as blood or milk, or through the aerosol route [12]. Bacteria of the genus *Brucella* are highly infectious, and doses as low as 10 to 100 bacteria are thought to be sufficient to cause the human disease [12]. Brucellae are therefore considered to be targets for the development of biological weapons and several countries were suspected of trying to weaponize *Brucella *species during the Cold War [12, 13]. The primary host for *Brucella abortus *is cattle where it leads to abortions causing significant economic losses [1]. As humans are not the primary hosts for brucellae, the most promising strategies to control and finally eradicate the disease seems to be through rigorous vaccination of its primary host, and efficient screening methods that can differentiate between vaccinated and infected animals [1]. However, most countries have not implemented an efficient program for disease control [1]. Prohibitive factors are most likely the costs involved but compared to the potential economic losses caused by animal brucellosis, these costs are negligible [1]. 

**Figure 1:**
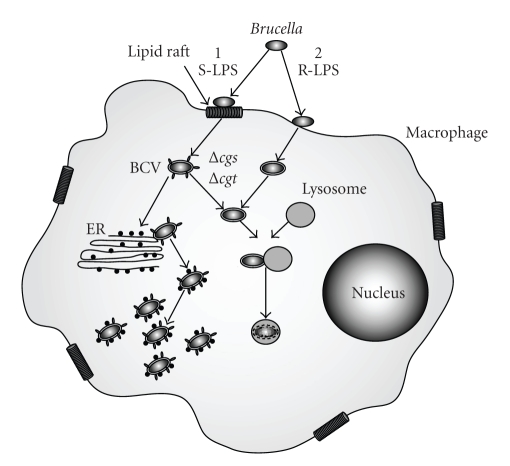
The *Brucella*-macrophage interaction. The preferred cells infected by *Brucella *are macrophages. *Brucella *strains with smooth LPS (S-LPS) enter the cell through interaction with lipid rafts and are then encompassed in a membrane bound compartment called *Brucella *containing vacuole (BCV). This vacuole retains some lipid raft markers, targeting the BCV to the endoplasmic reticulum (ER). *Brucella *fuses with the ER, thus acquiring ER markers to avoid fusion with the lysosome before beginning to replicate. Rough LPS mutants do not enter the macrophage through lipid rafts and are rapidly targeted to the lysosome and killed. Mutants in the C*β*G biosynthesis pathway (Δ*cgs *and Δ*cgt*) do not fuse with the ER but are targeted to the lysosome.

The infection process of *Brucella *has been subject to intense research. The preferred cell types infected by brucellae are phagocytic cells such as macrophages [14] (Figure 1). Brucellae are taken up into a phagosome, which is then targeted to the endoplasmic reticulum, the replicative niche of the *Brucella *within the host [12, 14]. Evasion of the immune system of the host organism and targeting of the bacterium to its replicative niche are of key importance for the infection process. The bacterial cell envelope is the major point of interaction between brucellae and the host and as such, molecules within the bacterial cell envelope play a significant part in the infection process. This paper will focus on the role of two brucellae sugar-modified cell envelope components, lipopolysaccharide (LPS) and cyclic *β*-1,2-glucans (C*β*Gs), in host interactions and vaccine development.

## 2. Lipopolysaccharide

**Figure 2:**
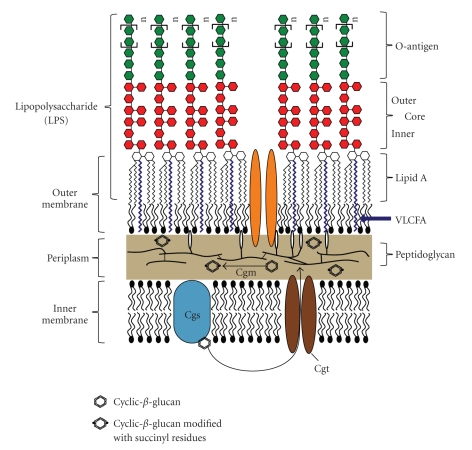
Diagrammatic representation of the *Brucella* cell envelope. The cell envelope is comprised of an inner membrane, consisting of a bilayer of phospholipids, and an outer membrane with an inner leaflet of phospholipids and an outer leaflet of lipopolysaccharide (LPS). LPS consists of three components. The O-antigen faces the extracellular space and it is the component that is recognized by the adaptive immune response. The O-antigen is connected to a sugar core molecule composed of different sugars which have not yet been fully identified. Lipid A forms the hydrophobic anchor of LPS within the membrane and has a backbone of diaminoglucose, which is acylated with saturated and hydroxylated fatty acids. *Brucella *lipid A contains an unusual very-long-chain fatty acid (VLCFA). Cyclic *β*-1,2-glucans are synthesized by the inner membrane protein Cgs and then transported to the periplasm by the predicted ABC-transporter Cgt where they are modified with on average two succinyl residues by a predicted membrane protein Cgm.

Brucellae are Gram-negative bacteria and as such their cell envelope is composed of two membranes (Figure 2). The outer membrane plays a crucial role in the infection process, as it is the first point of interaction between the bacterium and the host. The outer layer of the outer membrane is composed of LPS, which consists of three key components: (i) the lipid A, which forms the hydrophobic anchor of the LPS within the outer membrane, (ii) an inner and outer core composed of sugar molecules, and (iii) the O-antigen, a polymerized sugar chain extending into the extracellular environment (Figure 2). Brucellae occur naturally as smooth LPS (S-LPS) strains, which contain LPS that is modified with an O-antigen, and rough LPS (R-LPS) strains, which lack the O-antigen [15]. This paper will focus on the importance of *Brucella *LPS on the host interaction and on how its structure can influence the infection process. 

### 2.1. Importance of LPS for the Interaction with Host Cells

In many bacterial infections, LPS is the major molecule that is recognized by the innate immune system and can trigger a severe immune response against the invading organism. The ability of brucellae to produce S-LPS with a complete O-antigen is crucial for its virulence in humans [2]. *B. melitensis, B. suis, *and *B. abortus *that express a complete O-antigen are the main species responsible for human infections [2]. Mutants of these *Brucella *species lacking the O-antigen modification are considerably less virulent than their respective parent strains [15]. *Brucella *species that are naturally devoid of the O-antigen modification such as *B. canis *and *B. ovis* have either a low virulence or are avirulent in humans [2]. 

LPS is released by Gram-negative bacteria during their growth and death and is the cause of endotoxic shock in septic patients [16]. The LPS of *Brucella *is less toxic than that of enterobacterial species and therefore plays a major role for *Brucella* in the evasion of the host's immune system and its survival thereafter [17, 18]. LPS is the major surface antigen and is recognized by the immune system by the Toll-like receptor 4 (TLR4)/MD2 complex [19]. This complex binds the lipid A component of LPS and, by the recruitment of additional factors, initiates the innate immune response [19]. Although *Brucella *LPS binds to TLR4, it does not induce the production of cytokines and antimicrobial peptides [12]. *Brucella *LPS is several hundred times less effective at inducing the innate immunity than *E. coli *LPS, and this is thought to be important for *Brucella* to evade immune detection and to form a chronic intracellular infection [12, 15]. The LPS O-antigen plays an important role in the development of an adaptive immune response to pathogenic bacteria [19] and is the major antigen that is presented by the MHC II of B-cells [16]. However, *Brucella *LPS interacts with MHC II molecules in a way that prevents signaling and activation of MHC II dependent T-cells [16, 18]. Therefore, *Brucella *LPS acts as an important virulence factor and prevents the initiation of an adaptive immune response. The modification of *Brucella *LPS with its O-antigen also seems to be essential for the entry of the bacterium into the host cells (Figure 1). *Brucella abortus *strains having S-LPS enter the host cell via lipid rafts and thus the compartment in which they persist acquires lipid raft marker molecules that are important for the intracellular targeting of the compartment to the endoplasmic reticulum (Figure 1) [12, 14]. R-LPS *B. abortus *mutants do not enter the host cells through lipid rafts but through normal phagocytosis and are then targeted to lysosomes (Figure 1) [12, 14]. Therefore, LPS plays a crucial role for *Brucella *in evading both innate and adaptive immunity and in enabling the bacterium to reach its intracellular niche.

### 2.2. Structure and Biosynthesis

**Figure 3:**
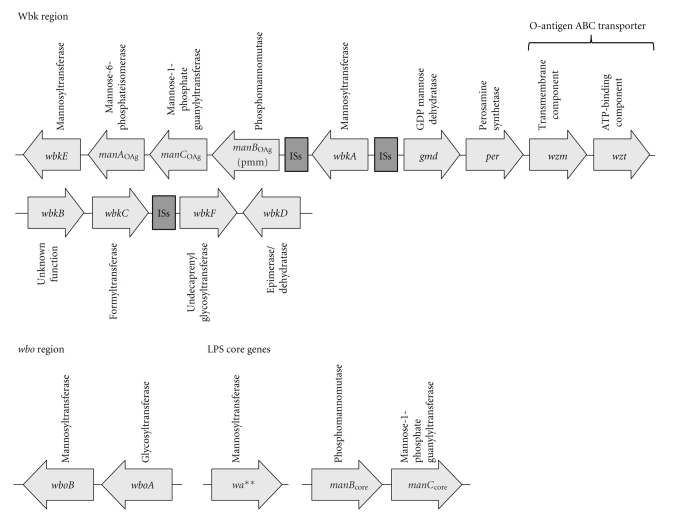
Genomic organization of O-antigen and core biosynthesis genes. The genes predicted to be involved in the biosynthesis of *B. melitensis *LPS O-antigen and core molecule are distributed at four different loci throughout the genome. The majority of the genes are located in the *wbk *region and the *wbo *region on chromosome I. Genes involved in the core biosynthesis can be found on chromosome I (*w* a**) and chromosome II (*m* *a* *n* *B*_core_ and *m* *a* *n* *C*_core_). The *wbk *region contains multiple insertion sequences (ISs) suggesting that this region was acquired through horizontal gene transfer [22].

**Figure 4:**
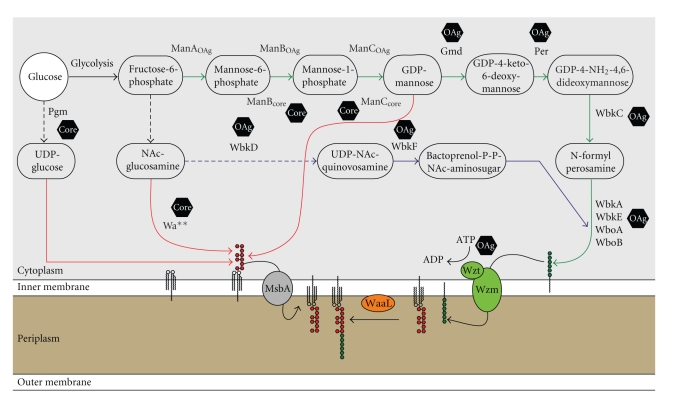
*Predicted pathways of LPS O-antigen and core molecule* biosynthesis. Brucellae derive all sugars from glucose. Green arrows indicate the reactions leading to the synthesis of the monomeric O-antigen subunits, which are polymerized onto bactoprenol (pathway indicated in blue). The complete O-antigen is then transported to the periplasmic face of the inner membrane by the ABC transporter system Wzm/Wzt and ligated to lipid A molecule by the O-antigen ligase WaaL. Pathways leading to the biosynthesis of the core molecule are indicated in red. Hexagons indicate whether mutants in the respective genes are affected in the biosynthesis of the O-antigen (OAg) or the sugar core molecule (Core). Dotted arrows indicate abbreviated pathways involving the defined enzymes.

*Brucella* only uses glucose as its carbon source and is therefore equipped with a set of enzymes to convert glucose into the different types of sugar molecules utilized within the organism (Figures 3 and 4) [20]. The biosynthesis of the *Brucella *O-antigen and core molecule has not yet been fully characterized and much has been derived from predicted protein functions and homologies to other microorganisms and from the identification of R-LPS mutant phenotypes (Figures 3 and 4) [20–24]. These data have been used to propose the biosynthetic pathway for the LPS O-antigen and core (Figure 4) [20]. 

The biosynthesis of the LPS core molecule and O-antigen is not located in a single region within the *B. melitensis *genome [23]. The majority of the O-antigen biosynthesis is located in the *wbk* region and an additional set of two glycosyltransferases in the *wbo *region on chromosome I (Figure 3) [23]. The *wbk *region has a lower G+C content (44%–49%) than the average G+C content of the entire *B. melitensis *genome (56%–58%) and is flanked by several insertion sequences indicating that it has been acquired by lateral gene transfer (Figure 3) [22]. The genes involved in the biosynthesis of the LPS core molecule are distributed on both *Brucella* chromosomes [23]. Two enzymes involved in the provision on mannose for the LPS core molecule are located on chromosome II whereas a putative glycosyltransferase involved in core biosynthesis is located on chromosome I [20, 22–24]. The glycosyltransferase region (*m* *a* *n* *B*_core_ & *m* *a* *n* *C*_core_) on chromosome II, however, was not acquired through lateral gene transfer but has evolved in the organism through a longer period as the G+C content of these genes is similar to the rest of the *Brucella *genome [24].

#### 2.2.1. Core Biosynthesis

The sugar core is linked to the O-antigen (Figure 2) and is composed of glucose, mannose, quinovosamine, glucosamine, 3-deoxy-D-manno-2-octulosonic acid (KDO), and several yet to be identified sugars residues [15]. As the full biochemical composition of the core molecule still remains to be determined, it is not yet possible to propose a biosynthetic pathway for the core sugar molecule of *Brucella *LPS. However, several mutants have been identified, that were defective in the correct synthesis of the LPS core.

**Phosphoglucomutase**

Pgm plays a central role in converting glucose-6-phosphate into glucose-1-phosphate, making it indispensable for the biosynthesis of many sugar molecules in brucellae (Figure 4) [25]. The *B. abortus *B2211 mutant was created by the insertion of a nonpolar gentamicin cassette into the *pgm* gene of *B. abortus *2308. A mutation in *pgm* causes a pleiotropic effect on the synthesis of oligo- and polysaccharides as Pgm is required for the production of ADP-glucose, UDP-glucose, and UDP-galactose, which themselves serve as sugar donors for later biosynthetic processes [25]. The *B. abortus pgm *mutant lacks the O-antigen, has an increased susceptibility to complement-mediated lysis relative to its parent strain, and is attenuated in its survival within a mouse model [26]. It is generally believed that the loss of the O-antigen makes bacteria more susceptible to complement-mediated lysis [15]. In addition to this, it was found to be involved in the biosynthesis of the LPS core molecule in *B. melitensis. *Here, the *pgm *mutant also lacked the O-antigen, but its LPS migrated lower on SDS PAGE gels suggesting a core defect (Figure 4) [20]. This observation is not surprising as glucose was identified to be a component of the sugar core of *Brucella *LPS [20, 26].

**Phosphomannomutase**

Pmm or ManB is encoded by the *B. abortus *and *B. melitensis pmm *and *manB *genes, respectively, [24] and is required for the conversion of mannose-6-phosphate to mannose-1-phosphate [15]. *Brucella *encodes two separate phosphomannomutase genes in its genome (Figure 3). While *manB* mutants in the *wbk* region do not have an impact on the O-antigen and core biosynthesis, mutants in the *m* *a* *n* *B*_core_ gene lack the O-antigen and are defective in the LPS core biosynthesis (Figures 3 and 4) [20, 22–24]. The same observation has been made for the *manC *genes in the *wbk *region, which does not seem to influence either O-antigen or core biosynthesis and the *m* *a* *n* *C*_core_ gene on chromosome II (Figures 3 and 4) [20, 22–24]. The *manC *genes are predicted to encode mannose-1-phosphate guanylyltransferases required for nucleotide activated mannose-1-phosphate provision (Figure 3) [20, 22–24]. It has been hypothesized that the *m* *a* *n* *B*_core_ and *m* *a* *n* *C*_core_ genes can provide mannose both for the biosynthesis of the O-antigen and the core molecule, whereas the *manB* and *manC* genes in the *wbk* region only provide mannose for the O-antigen biosynthesis [20, 22–24]. This assumption is reasonable as the *m* *a* *n* *B*_core_ and *m* *a* *n* *C*_core_ genes have not been acquired by lateral gene transfer as were the majority of the genes involved in the O-antigen biosynthesis in the *wbk *region. It is therefore possible that *m* *a* *n* *B*_core_ and *m* *a* *n* *C*_core_ have evolved primarily for the provision of mannose to the core biosynthesis pathways but can also provide mannose for the O-antigen polymerization. Conversely, the *manB *and *manC *genes in the *wbk *region have evolved to provide mannose for the O-antigen biosynthesis and cannot compensate for the loss of the *m* *a* *n* *B*_core_ and *m* *a* *n* *C*_core_ genes in the core biosynthesis.

The *w* a** Gene

This gene encodes a putative glycosyl transferase in the *Brucella *genome. A mutant in the *w* a** gene was isolated from screening a transposon mutant library for polymyxin-sensitive mutants [24]. Polymyxin is a cationic peptide that acts by perturbing membranes of bacteria by binding to LPS and increasing the permeability of the cell envelope, and mutants affected in their sensitivity to polymyxin are likely to be affected in the LPS molecule [27]. The transposon insertion in the *w* a** mutant disrupts a putative glycosyl transferase gene, which is thought to be involved in the biosynthesis of the LPS core (Figures 3 and 4). The *B. abortus* *w* a** mutant retains full reactivity with antibodies directed against the outer core epitope but has a reduced reactivity for antibodies specific for the inner core epitope, suggesting that *w* a** is required for the correct synthesis of the inner core of the *Brucella abortus *LPS (Figures 2 and 3) [24].

#### 2.2.2. O-Antigen Biosynthesis

The O-antigen of *B. abortus *is composed of a linear homopolymer that consists of *α*-1,2-linked 4,6-dideoxy-4-formamido-*α*-D-mannopyranosyl subunits with a chain length between 96 and 100 subunits [15, 22]. O-antigen homopolymers, in contrast to heteropolymers, are synthesized via a Wzy-independent mechanism and after its assembly at the cytoplasmic face of the inner membrane onto bactoprenol phosphate, the complete lipid-linked O-antigen is transported across the inner membrane using an ATP-binding cassette (ABC) transporter system [20, 22]. Wzm and Wzt were identified and predicted to form the transmembrane and ATPase domain, respectively, of an ABC transporter required for the translocation of the full length homopolymer O-antigen from the cytoplasmic to the periplasmic face of the inner membrane (Figures 3 and 4) [22]. Indeed, the deletion of *wzm*/*wzt *resulted in the accumulation of intracellular O-antigen further supporting this hypothesis [22]. Mutants in the biosynthetic pathways involved in the provision of perosamine or bactoprenol-P-P-NAc-aminosugars encoded in the *wbk *and *wbo *regions on chromosome I are all affected in the formation of the *Brucella *O-antigen (Figure 4 blue and green pathways) [20–22, 24, 28].

#### 2.2.3. Genetic Variation among *Brucella* Species

A recent study has analyzed DNA polymorphisms in *wbkE*, *manA*, *manB*, *manC*, *wbkF, wkdD, wboA, wboB,* *w* a**, and *m* *a* *n* *B*_core_ genes between different naturally smooth and rough *Brucella *species [23]. Interestingly, it was found that *B. ovis*, a strain producing naturally rough LPS, lacked the *wbo *region and the *manA* gene but was otherwise identical to smooth LPS strains [23]. *B. canis, *another naturally rough LPS strain, had a deletion in *wbkD *and also in *wbkF*, both required for the correct O-antigen synthesis [23]. Interestingly, only very little polymorphisms were found for the *m* *a* *n* *B*_core_, *m* *a* *n* *C*_core_, and *w* a** genes involved in the core biosynthesis [23]. This is in agreement with a conserved structure in the LPS core of brucellae and also with a long coevolution of the LPS core biosynthesis genes among brucellae.

### 2.3. Lipid A

The lipid A backbone of *Brucella *is composed of 2,3-diamino-2,3-dideoxy-D-glucose. This sugar backbone is modified with saturated fatty acids ranging from C_16:0 _to C_18:0 _and hydroxylated fatty acids ranging between 3-OH-C_12:0_ to 29-OH-C_30:0_ [15]. The unusual modification of *Brucella *LPS with very-long-chain fatty acids (VLCFA) has been implicated to be of key importance in stabilizing the *Brucella *membrane to the conditions encountered within the host. A *B. abortus bacA *mutant has a reduction in the VLCFA-content of its LPS [29] and was defective in its chronic infection in BALB/c mice [30]. In the phylogenetically related bacterium, *Sinorhizobium meliloti*, a *bacA *mutant was also defective in forming an interaction with the plant host, alfalfa [31]. The lipid A of *S. meliloti* is also modified with a VLCFA and deletion of the *bacA *gene resulted in a reduction in the lipid A VLCFA-content [29, 32]. However, *S. meliloti* mutants in the biosynthesis pathway of these lipid A VLCFA modifications still formed a successful alfalfa interaction but were substantially reduced in their competitiveness relative to the parent strain [33, 34]. *S. meliloti* mutants lacking the lipid A VLCFA modification also showed defects in the development of nitrogen-fixing bacteroides [35]. Taken together, these findings indicate that the modification of lipid A with VLCFA plays an important role in the interaction of *α*-proteobacteria with their hosts. It has been proposed that the lipid A VLCFA is important for either protecting bacteria from the conditions encountered within the host cells, that is, by stabilizing their outer membrane and/or in subverting the immune or defense response of the host [29, 32].

## 3. Cyclic-*β*-1,2-Glucans

### 3.1. Biosynthesis

Cyclic *β*-1,2-glucans (C*β*Gs) are polymers of 17 to 24 *β*-1,2-linked cyclic glucose molecules. *Brucella* species belong to the same category of *α*-proteobacteria as *Agrobacterium tumefaciens *and *S. meliloti*, which also produce C*β*Gs [36]. As in other *Rhizobiaceae*, the biosynthesis of C*β*Gs in *Brucella *is catalyzed by a single cyclic glucan synthase, Cgs (known as NdvB and ChvB in *S. meliloti *and *A. tumefaciens*, respectively) that can facilitate the four enzymatic reactions: (i) initiation, (ii) elongation, (iii) phosphorolysis, and (iv) cyclization required for the synthesis of C*β*Gs [37–42]. Cgs uses UDP-glucose as a sugar donor and transfers the glucose molecule onto an unknown amino acid of Cgs, which acts as an intermediate for the synthesis of C*β*G (Figure 2). The linear glucan chain is extended to a final length of 17 to 24 glucose molecules before it is cyclized [39]. The degree of polymerization of C*β*Gs is determined by a C-terminal domain of Cgs [43]. After their synthesis, the C*β*Gs are transported into the periplasm by an ABC-transporter system involving the Cgt protein, which contains conserved motifs of ATP-binding proteins (Figure 2) [44]. In the periplasm, *Brucella *C*β*Gs are then modified with on average 2 O-ester-linked succinyl residues per molecule by a protein called cyclic glucan modifier (Cgm) (Figure 2) [45].

### 3.2. Regulation

Based on comparative studies with the closely related organisms, *A. tumefaciens *and *S. meliloti, *it was hypothesized that C*β*G expression in *Brucella *could be osmo-regulated as was the case for the aforementioned bacterial species [37]. Gram-negative bacteria accumulate osmolytes in their periplasm in order to adapt to changes in the osmolarity of their environment [46]. C*β*Gs biosynthesis in *S. meliloti *and *A. tumefaciens *has been shown to be involved in the adaptation of these bacterial species to hypo-osmotic conditions and NdvB and ChvB mutants, in the cyclic-*β*-glucan synthases of *S. meliloti *and *A. tumefaciens*, respectively, are unable to produce C*β*Gs when grown in media with a low osmotic pressure [37]. The accumulation of cellular C*β*Gs was also inhibited when grown under high osmotic conditions [46]. Therefore, *in-vivo* data suggested that C*β*G expression is essential for the adaptation of *A. tumefaciens *and *S. meliloti *to low osmotic pressures and was down-regulated under high osmotic conditions. However, membrane extracts of *S. meliloti *and *A. tumefaciens *showed no inhibition of C*β*G biosynthesis at high mannitol or sucrose concentrations (up to 400 mM) suggesting that the high osmolarity itself was not responsible for the reduced C*β*G accumulation *in-vivo* [37]. When the membranes were incubated with high NaCl or KCl concentrations (starting from less than 100 mM NaCl or KCl, respectively), the accumulation of C*β*Gs was significantly reduced when high osmolarity was achieved by the addition of sodium chloride or potassium chloride [37]. The intracellular accumulation of potassium ions followed by glutamate biosynthesis is a major response of Gram-negative bacteria to osmotic up-shock and the bacteria prevent dehydration by the acquisition of compatible solutes [47]. Hence it is possible that the uptake of KCl and the production of other osmolytes *in-vivo* inhibit the accumulation of C*β*Gs in environments with high osmotic pressures. 

 C*β*G biosynthesis in *Brucella *species, however, is not osmo-regulated [38]. *B. abortus *S19, an attenuated strain of the virulent strain 2308 and *B. ovis *REO198, showed no reduction in cellular C*β*G accumulation when grown under high osmotic conditions [38]. *B. abortus *S19 membrane extracts were also found not to be inhibited in the biosynthesis of C*β*Gs when exposed to high KCl concentrations [40]. When the *B. abortus cgs *gene was introduced in *A. tumefaciens *and *S. meliloti *mutants in the cyclic glucan synthase genes, *chvB *and *ndvB, *respectively, membranes extracted from these bacterial strains carrying the *B. abortus cgs *gene were able to synthesize C*β*Gs even at high KCl concentrations of 250 to 500 mM KCl [40]. Conversely, when the *A. tumefaciens chvB *gene was introduced into a *B. abortus cgs *mutant, it restored its ability to produce C*β*Gs but the complemented strain was unable to incorporate [^14^C] glucose when incubated under high potassium glutamate conditions [40]. Therefore, *Brucella *cyclic glucan synthase, in contrast to the *Agrobacterium *and *Sinorhizobium *enzymes, is not inhibited by the acquisition of intracellular osmolytes in response to osmotic up-shifts. However, it is interesting to note that, when a *B. abortus *S19 *cgs *mutant was complemented with the *A. tumefaciens chvB *gene, the *in-vivo* acquisition of C*β*Gs was not affected by high osmolarity [40]. This suggests that an alternate mechanism is present in *Brucella *that can protect the enzyme from the inhibition by osmolytes. Glycine betaine has been shown to protect and stabilize enzyme function under hyperosmotic conditions, and the function of *A. tumefaciens *and *S. meliloti *membrane extracts to produce C*β*Gs under these conditions could partially be recovered by the addition of glycine betaine [40]. In fact, the osmoregulation of C*β*G production might not be required in *Brucella*, which are poorly adapted to survival outside a host cell and therefore naturally reside within an iso-osmotic environment.

### 3.3. Role in Mice Infections

It was shown that, relative to the parent strain of *Brucella abortus *2308 (a virulent strain) or *B. abortus *S19 (an attenuated strain used for vaccination), mutants in the cyclic glucan synthase gene *cgs *and in the cyclic glucan transporter gene *cgt *were recovered at a decreased amount from the spleens of infected mice [38, 39, 44]. Mice infected with either the *B. abortus* 2308 parent strain, *cgs *or *cgt* mutant strain, showed no difference in the number of colony forming units (CFUs) recovered from spleens 4 weeks postinfection [44, 48]. However, after 8 weeks, the number of CFUs in the *B. abortus *2308 *cgt *and *cgs *mutants was reduced by approximately 10- to 100- fold, respectively, while levels in mice infected with the parental and complemented mutant strains stayed the same [44]. The rate at which the bacteria were “cleared” from infected mice was significantly different between the virulent strain of *B. abortus *2308 and the vaccine strain S19 [44]. In the *B. abortus *S19 background, the *cgs *and *cgt *mutant showed significant losses of recoverable *Brucella *relative to the parent and complemented strain of 100- to 1000- fold, two weeks postinfection [44]. Eight weeks postinfection, the *B. abortus *S19 *cgs *mutant was virtually cleared from the infected mice spleens [48]. At this time point, no difference was observed in mice infected with the *B. abortus *2308 parent and *cgs *mutant strains [48]. C*β*G therefore plays a major role in the persistence of Brucella within the host environment. It is noteworthy that, despite being less virulent, the *B. abortus *S19 *cgs *mutant retained its ability to confer protection in mice against subsequent *B. abortus *2308 infections to a similar degree as the S19 vaccine strain [48].

### 3.4. Role in Intracellular Replication

The infection process of *Brucella *can be grouped into two phases: the invasion phase (0–8 hours postinfection) during which *Brucella *enters the host cells but does not yet replicate, and the replication phase when *Brucella* has reached the rough endoplasmic reticulum and starts to replicate (Figure 1) [48]. In infection models using HeLa cells, it was shown that *B. abortus *2308, *B. abortus *S19, and their respective *cgs *mutants were not affected during cell invasion as the same number of CFUs was recovered 4 hours postinfection [48]. However, *cgs *mutants in both *Brucella *strains showed a lower rate of intracellular replication relative to the respective parent strains suggesting that C*β*Gs are required for the normal replication of *Brucella *within the host cell [48]. This phenotype has also been described for a mutant in the *pgm *gene [26]. This is not surprising, as mentioned earlier (Section 2.2), Pgm is essential for the provision of UDP-glucose, the sole sugar donor for C*β*G biosynthesis [49]. 

A recent study showed that C*β*Gs have similarities to cyclodextrins, cyclic oligosaccharides consisting of *α*-1,4-linked glucopyranose units [50]. Cyclodextrins contain a lipophilic cavity, which allows them to extract cholesterol from membranes [50]. Cholesterols are a major component of lipid rafts, microdomains on eukaryotic cell membranes, which have an increased density of sphingolipids and cholesterol [50]. These lipid rafts are involved in the selective transport of molecules, can serve as relay stations for intracellular signaling and play an important role as attachment sites for toxins and pathogens [50]. Lipid rafts can also be found intracellularly on phagosomes and have been proposed to be involved in phagosome maturation. Therefore, phagosomal lipid rafts present an ideal target for intracellular pathogens, which could influence intracellular signaling and/or trafficking by modifying phagosomal lipid raft domains [50]. 

Brucellae need to enter the host cell via these lipid raft domains, in order to establish a successful and persistent infection (Figure 1). C*β*Gs have been shown to be able to perturb eukaryotic cell membranes and to extract cholesterol from lipid rafts, suggesting that they might work in a similar manner to cyclodextrins [50]. The role of C*β*Gs in the virulence of *B. abortus *has been discussed for a long time The conclusive proof that C*β*Gs, and not pleiotropic effects originating from the disruption of C*β*G, are the cause for the defects in the interaction of *Brucella *with its host was shown recently [50]. By adding external C*β*Gs to cell cultures infected with *B. abortus cgs *mutant strain, they were able to restore its ability to replicate within host cells to the level of the parent strain [50]. Inside the host cell, *Brucella *manipulate the phagosome to evade lysosome fusion and target the *Brucella *containing vacuole (BCV) to the endoplasmic reticulum (Figure 1) [50].

 C*β*Gs play a crucial role in the process of evading the fusion with the lysosome and directing the BCV to the endoplasmic reticulum. The BCV of cells infected with the *B. abortus cgs* mutant progressively acquired cathepsin D, a lysosomal hydrolase, while in cells infected with C*β*G-treated *B. abortus cgs *mutant and the *B. abortus *parent strain 2308, the *Brucella* strains were able to replicate within cathepsin D negative BCVs (Figure 1) [50]. Similar results were obtained when monitoring the acquisition of endoplasmic reticulum-marker proteins such as calreticulin to the BCV and only BCV with the parent strain and the C*β*G-treated *cgs *mutant strain were able to fuse with the ER (Figure 1) [50]. C*β*Gs are therefore an important virulence factor in *Brucella *infections, enabling the bacteria to modify the lipid raft domains of the BCV to avoid lysosome fusion and to target the BCV to the endoplasmic reticulum [50]. C*β*Gs seem to be important throughout the whole infection process, and it has been suggested that because of the key role of C*β*G it might be crucial for *Brucella *that C*β*G biosynthesis is not osmoregulated and can be expressed at sufficient levels throughout [50].

### 3.5. Cyclic-*β*-Glucans in Other *α*-Proteobacteria

C*β*Gs are found among many *α*-proteobacteria and have been determined to play a crucial role for *Rhizobiaceae *to interact with their host [38]. *S. meliloti *and *A. tumefaciens* cyclic glucan synthase mutants in the *ndvB *and *chvB *genes, respectively, were unable to synthesize C*β*Gs and were incapable to invade their host plants to form nitrogen fixing nodules or to cause tumor formation, respectively [38, 51]. It was therefore hypothesized that C*β*Gs play a major role for the host infection of these bacteria with their hosts. Cyclic glucan synthase is a large inner membrane enzyme and *A. tumefaciens, *and *S. meliloti *mutants defective in this enzyme show pleiotropic membrane defects such as increased sensitivity to antibiotics and detergents and are nonmotile due to an inability to assemble flagella [48]. An *A. tumefaciens chvB *mutant has also been shown to be defective in the attachment to surfaces as it produced an inactive form of the protein rhicadhesin [52] and to express lower level of the VirB10 transmembrane protein that is part of a type IV secretion system required for the virulence of *A. tumefaciens *and *Brucella *species [53]. Due to these pleiotropic defects of cyclic glucan synthase mutants, the role of C*β*Gs in the virulence of these organisms was uncertain.

## 4. Vaccination Strategies against *Brucella * Species

As most brucellosis cases are contracted through contact with infected animals or their products, eradication strategies for the disease are mainly focused on eliminating *Brucella*, the causative agent of brucellosis in its primary host [12]. Live, attenuated strains and dead *Brucella *are used for the vaccination of animals, and this strategy has been employed successfully in a number of countries to eradicate the disease [12]. In the following subsections, we will discuss several mutants that were or are used for the vaccination of animals against brucellae.

### 4.1. *B. abortus* S19

The most commonly used strain to vaccinate cattle is *B. abortus *S19 [54] and a derivative of it has been used in the former USSR as a live vaccine for humans [55]. However, *B. abortus *S19 is not entirely avirulent in humans and cases have been reported in which veterinarians dealing with the vaccine strain for the immunization of cattle have become infected [2]. *B. abortus *S19 was originally isolated from milk of an infected animal as a virulent strain, but has become attenuated by a spontaneous, unknown mutation during laboratory culturing [56]. Despite being attenuated, it is serologically indistinguishable from virulent strains and produces S-LPS [57]. The effectiveness of vaccinations with this strain and the production of antibodies to it are dependent on the age of the animal at the vaccination, the dose, and the route that the vaccine is applied and the prevalence of brucellosis within the herd [54]. After the animal is vaccinated, it will be protected from brucellosis for several years, which can then be extended by revaccination [54]. However, *B. abortus *S19 is not completely avirulent for cows and it can cause abortions in a small percentage (<2.5%) of immunized, pregnant cows and orchitis in bulls [54, 58]. Therefore, vaccination with *B. abortus *S19 is currently limited to female calves between the age of three to eight months [59]. The *B. abortus *S19 vaccine strains have also been linked to arthropathy, when S19 antigen-containing immune complexes were found in joints of brucellosis free but vaccinated cattle [60]. A recent study has identified that mutations in 24 genes of *B. abortus* S19 relative to *B. abortus* 2308 that may account for its loss of virulence [61]. Among these 24 genes, some were encoding proteins involved in the metabolism of erythritol and lipids [61].

### 4.2. *B. melitensis* Rev.1

*B. melitensis *Rev.1 is the most efficient vaccine strain used to immunize sheep and goats [58]. The strain was derived from a virulent strain that was developed to be used as a life vaccination strain and was made dependent on streptomycin for its growth to control it within the host [62]. However, although this strain conferred protection in mice against brucellosis, it proved inefficient at protecting monkeys and goats [62]. Therefore, a strain was selected from isolates that were successful at immunizing mice and guinea pigs that was no longer dependent on streptomycin and had also lost some of its virulence [62]. *B. melitensis *Rev.1 is used as a live vaccine and as it retains some virulence will lead to abortions if used on pregnant animals [58, 62]. *B. melitensis *Rev.1 has been reported in rare cases to be excreted into the milk of lactating animals, raising concerns about the vaccination strain infecting other animals and humans [62]. In fact, cases of animal abortions caused by *B. melitensis *infections and human brucellosis have been reported in which *B. melitensis *strains were isolated that were identical in their appearance to the Rev.1 vaccination strain but were sensitive to streptomycin [62]. *B. melitensis *Rev.1 has an S-LPS phenotype and animals vaccinated with this strain will raise antibodies against the O-antigen of *B. melitensis* [58]. This makes distinguishing between vaccinated and infected animals using serological laboratory methods virtually impossible [58].

### 4.3. *B. abortus* Strain 45/20

*B. abortus *45/20 is an R-LPS mutant that has been obtained after 20 passages of the *B. abortus *isolate 45 through guinea pigs [58, 63]. This mutant strain produces a small amount of O-polysaccharide, which is polymerized in a different way to the wild type strains and contains a reduced number of sugar units [64]. Although this strain was able to protect guinea pigs from *Brucella *infections, it is not a stable live vaccine and can revert back into a virulent strain [58]. However, when used in the vaccination of cattle, it did not cause abortions [58]. As the mutation causing the R-LPS phenotype of *B. abortus *45/20 is unknown [58] and reversion to the S-LPS and virulent strain can occur, it is not currently used as a vaccine strain [63].

### 4.4. *B. abortus* RB51

*B. abortus *RB51 is an attenuated spontaneous R-LPS mutant that was obtained by repeated passage of *B. abortus *2308 on media containing rifampicin and penicillin [65]. RB51 carries an IS771 insertion in *wboA*, a gene encoding a putative glycosyl transferase, and is thought to have several other unknown mutations [28]. Even though RB51 has a mutation in the *wboA *gene, its LPS contains 2.5 times less mannose than that of other *B. abortus wboA *mutants [28]. In contrast to 45/20, this strain was shown to be a stable rough mutant even after multiple passages through laboratory cultures and through infected animals [65]. Therefore, it is currently used in several countries as a vaccine for cattle [66]. RB51 has been shown to be efficient in protecting mice against brucellosis but was not effective when tested in sheep [58]. RB51 is resistant to rifampicin, which is used in the treatment of human brucellosis [63].

### 4.5. Advantages and Disadvantages of Vaccination Strategies

Many other R-LPS *Brucella *strains are available for vaccine development (refer to Section 2). However, as the O-antigen is the most exposed antigen during *Brucella *infections the majority of the adaptive immune response is targeted towards this antigen. Therefore, rough *Brucella *mutant strains in general confer a lower degree of protection than S-LPS strains. Live attenuated strains of *Brucella *are the current method of choice for the vaccination of animals against *Brucella *infections; however, there is currently no licensed human vaccine available [55]. The increased level of protection conferred by attenuated S-LPS strains is preferable to the lower level obtained by R-LPS *Brucella *strains. On the other hand, S-LPS vaccine strains are bound to cross-react in the diagnostic assay used to determine *Brucella *infections [55] and will therefore make it impossible to distinguish between vaccinated and infected animals [55]. Finding an attenuated *Brucella *strain that is both effective in raising a sufficient protection from subsequent infections and that does not interfere with standard diagnostic test is a challenge for the future, and several candidates have been determined in screens for *Brucella *mutants that are attenuated in their virulence [55].

## 5. Conclusions

Sugar-modified cell envelope components play a central role in the interaction of *Brucella *with their hosts. LPS is a key mediator that is detected by the innate immune response. It is therefore not surprising that *Brucella *LPS is more adapted to circumvent the activation of the host's innate immunity. The unusual structure of *Brucella *LPS contributes to suppressing as well as protecting the bacterium from the immune response of the host. C*β*Gs are important for intracellular replication, phagosome maturation, lysosome evasion, and targeting of *Brucella *containing vacuoles to the endoplasmic reticulum. This is achieved by modulating lipid raft domains of the host cell membrane and the membranes of BCVs. To facilitate this, constitutive expression of C*β*Gs is required and this could explain the lack of osmo-regulation found in the biosynthesis of *Brucella *C*β*Gs. C*β*Gs are produced by a number of *α*-proteobacteria that form interactions with eukaryotic host cells and were found to be essential for host interactions. This suggests that C*β*Gs are a key factor for repressing the perception of these different bacterial species by their hosts. This paper highlights the importance of both the LPS and C*β*Gs for *Brucella *to persist within eukaryotic cells. Therefore, sugar-modified cell envelope components are key virulence factors for *Brucella-*host infections. *Brucella* mutants that are either defective or result in changes to these sugar-modified components are often attenuated in their hosts. It will be interesting to determine whether these mutants can be developed in the future as vaccines to protect both animals and humans against *Brucella* infections.
